# Hospital, Emergency Department, and Home Health Use in Older Adults with Sensory Impairment

**DOI:** 10.1093/geroni/igab046.3612

**Published:** 2021-12-17

**Authors:** Laura Wallace, Karen Hirschman, Mary Naylor, Liming Huang, Pamela Cacchione

**Affiliations:** 1 University of Pennsylvania, Philadelphia, Pennsylvania, United States; 2 University of Pennsylvania, University of Pennsylvania, Pennsylvania, United States; 3 University of Pennsylvania, University of Pennsylvania School of Nursing, Pennsylvania, United States

## Abstract

Hearing, vision, and dual (combined hearing and vision) sensory impairments (HI, VI, and DSI) are common in older adults and associated with adverse health outcomes. However, it is not clear how sensory impairments impact healthcare utilization in older adults. This study aims to examine hospital, emergency department (ED), and home health care use amongst adults 65 and older diagnosed with HI, VI, and DSI in an urban academic health system. This secondary analysis (N=45,000) used a limited data subset of older adult primary care patients’ EHR data from a parent study examining medical complexity, healthcare use, and social vulnerability. Using logistic regression and controlling for participant demographics and comorbidities, results show HI, VI, and DSI increase the likelihood of having an ED visit (OR 1.29, p<.0001; OR 1.28, p=0.0011; OR 1.50, p=.0328, respectively) and a home health episode (hearing OR 1.41, p<.0001; vision OR 1.42, p=.0002) compared to those without sensory impairment (SI). No significant difference was found in hospital use and home health use for DSI. This is the first known study to examine ED use for older adults with VI and DSI, and home health use for older adults with SI in the US. Findings suggest older adults with SI have greater utilization and dependence on healthcare services. Older adults with SI may benefit from outpatient assessments and interventions to mitigate risks of ED use. Findings also support research into the drivers of healthcare use amongst this population, financial implications, and intervention development to prevent avoidable healthcare use.

